# Clinical, psychopathological, and biological predictors of resumption of menses in subjects with anorexia nervosa: A 4-year follow-up study

**DOI:** 10.1192/j.eurpsy.2021.330

**Published:** 2021-08-13

**Authors:** E. Rossi, E. Cassioli, G. Castellini, L. Giardinelli, A. Fanelli, A. Fisher, L. Vignozzi, V. Ricca

**Affiliations:** 1 Psychiatry Unit, Department Of Health Sciences, University of Florence, Florence, Italy; 2 Central Laboratory, Azienda Ospedaliero-Universitaria Careggi, Florence, Italy; 3 Andrology, Women’s Endocrinology And Gender Incongruence Unit, Department Of Experimental, Clinical, And Biomedical Sciences, University of Florence, Florence, Italy

**Keywords:** anorexia nervosa, Amenorrhea, resumption of menses, childhood abuse

## Abstract

**Introduction:**

Amenorrhea is one of the most frequent and serious consequences of Anorexia Nervosa (AN). Resumption of menses (ROM) is considered an important goal and is associated with a better outcome.

**Objectives:**

To investigate the role of age, Body Mass Index (BMI), diagnostic subtype (restrictive vs binge-purging), history of childhood abuse, duration of illness, psychopathology and sex hormones on ROM in AN.

**Methods:**

52 patients with AN and amenorrhea were enrolled at the start of treatment. Clinical parameters of interest were collected, and questionnaires were administered for the assessment of general (SCL-90-R) and specific (EDE-Q) psychopathology. Blood samples were taken to assess FSH, LH and estradiol levels. All patients were monitored regularly through psychiatric checkups until ROM, for up to four years.

**Results:**

A total of 30 (57.7%) subjects recovered their menstrual cycle in the follow-up period (mean time: 18.7 ± 14.8 months). Recovery was more frequent in the binge-purging subtype than in the restrictive subtype (82.4% vs 48.6%, p=0.019), and was significantly associated with diagnostic crossover (odds ratio=10.0, p=0.032). Multivariate Cox regression showed an increased likelihood of menstrual recovery for binge-purging subtype (p=0.005) and for those reporting a history of childhood abuse (p=0.025). Early ROM was also associated with baseline SCL-90-R scores (p=0.002) and FSH (p=0.011), while a longer duration of illness (p=0.003) and EDE-Q scores (p=0.009) predicted a later recovery.

**Figure:**
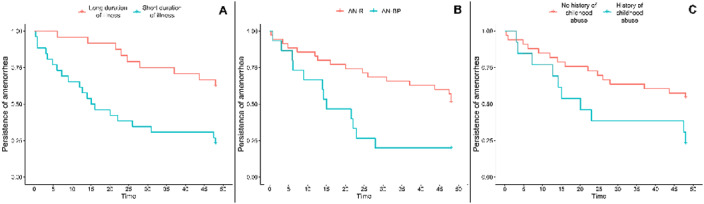

**Conclusions:**

This study highlights the role of duration of illness, childhood abuse history and psychopathological characteristics in subjects with AN at the start of treatment in predicting ROM.

**Disclosure:**

No significant relationships.

